# A low-cost, highly functional, emergency use ventilator for the COVID-19 crisis

**DOI:** 10.1371/journal.pone.0266173

**Published:** 2022-03-30

**Authors:** Samuel J. Raymond, Sam Baker, Yuzhe Liu, Mauricio J. Bustamante, Brett Ley, Michael J. Horzewski, David B. Camarillo, David N. Cornfield

**Affiliations:** 1 Department of Bioengineering, Stanford University, Stanford, CA, United States of America; 2 Department of Comparative Medicine, Stanford University, Stanford, CA, United States of America; 3 Department of Electrical Engineering and Computer Science, UC Berkeley, CA, United States of America; 4 Kaiser Pulmonology and Critical Care, Fontana, CA, United States of America; 5 O2U Inc., Stanford, CA, United States of America; 6 Department of Mechanical Engineering, Stanford University, Stanford, CA, United States of America; 7 Department of Neurosurgery, Stanford University, Stanford, CA, United States of America; 8 Department of Pediatrics—Pulmonary Medicine, Stanford University, Stanford, CA, United States of America; Yale University, UNITED STATES

## Abstract

Respiratory failure complicates most critically ill patients with COVID-19 and is characterized by heterogeneous pulmonary parenchymal involvement, profound hypoxemia and pulmonary vascular injury. The high incidence of COVID-19 related respiratory failure has exposed critical shortages in the supply of mechanical ventilators, and providers with the necessary skills to treat. Traditional mass-produced ventilators rely on an internal compressor and mixer to moderate and control the gas mixture delivered to a patient. However, the current emergency has energized the pursuit of alternative designs, enabling greater flexibility in supply chain, manufacturing, storage, and maintenance considerations. To achieve this, we hypothesized that using the medical gasses and flow interruption strategy would allow for a high performance, low cost, functional ventilator. A low-cost ventilator designed and built-in accordance with the Emergency Use guidance from the US Food and Drug Administration (FDA) is presented wherein pressurized medical grade gases enter the ventilator and time limited flow interruption determines the ventilator rate and tidal volume. This simple strategy obviates the need for many components needed in traditional ventilators, thereby dramatically shortening the time from storage to clinical deployment, increasing reliability, while still providing life-saving ventilatory support. The overall design philosophy and its applicability in this new crisis is described, followed by both bench top and animal testing results used to confirm the precision, safety and reliability of this low cost and novel approach to mechanical ventilation. The ventilator meets and exceeds the critical requirements included in the FDA emergency use guidelines. The ventilator has received emergency use authorization from the FDA.

## Introduction

COVID-19 is highly contagious and can lead to respiratory distress, severe hypoxemia and respiratory failure [1]. The World Health Organization estimates that 1 in 5 adults who contract the disease will require hospitalization for breathing difficulties, and 1 in 20 will receive care in the Intensive Care Unit (ICU) for respiratory failure and require mechanical ventilation [2]. With the likelihood that the virus will continue to mutate, challenges related to disease transmission, prevention and treatment of COVID-19 will persist for many years to come [3]. In the presence of a highly contagious and virulent virus, as is the case in the present pandemic, the need for hospital care, specialized equipment, and skilled care providers, even in resource replete countries, can far outpace capacity. With limited equipment and skilled personnel, care is compromised and both morbidity and mortality increase substantially. During the COVID-19 pandemic, communities across the globe have confronted profound resource shortages and devastating loss of life. Medical personnel have been forced to make decisions about which lives most merited ongoing life support. During the early months of the pandemic, in northern Italy, New York City, and areas of South America, shortages of equipment prompted care givers to withhold care for patients with a low statistical probability of survival, especially for many patients over 60 years of age [4, 5].

Mechanical ventilators, devices that facilitate both oxygenation and ventilation, first became widely available in the 1950s. Initially, mechanical ventilators used time-cycled negative pressure to facilitate gas exchange, technology that addressed the respiratory insufficiency due to muscular weakness resulting from infection with the polio virus. Subsequently, with more respiratory failure resulting from lung injury, positive pressure ventilation became more standard [6]. As more insight into the pathophysiology of lung injury has accrued, superimposed on rapid advances in engineering, mechanical ventilators have become ever more sophisticated, expensive and maintenance intensive. The most commonly deployed ventilators in United States can be set to deliver gas in multiple modes, include a minimum number of alarms and alerts, require hours of testing prior to being deployed in the clinical arena, with a cost approaching 50,000 USD per unit [7].

Given the profound shortage of resources, especially mechanical ventilators, our group, like many others [8], sought to address the issue by creating a safe, low-cost, rapid to manufacture and deploy ventilator, with sufficient functionality to provide both non-invasive and invasive mechanical ventilation. Our goal was to create a device that could be safely stored over the long-term with the capacity for a rapid quality control check and ready deployment into the patient care arena. With rapid changes in patient condition, superimposed on the potential for highly contagious pathogens, our intent was to design a ventilator capable of continuously delivering oxygen-rich air at flow rates as high as 60 liters per minute as used in high-flow nasal cannula and CPAP devices and positive pressure ventilation in both assisted and intermittent mandatory modes. To enable rapid and high-volume manufacturing, the ventilator design minimized the absolute number of parts.

To address the critical shortage of mechanical ventilators, the FDA provided detailed information surrounding the process and requirements for devices eligible for Emergency Use Authorization (EUA) which limited requirements to those vital to health and safety requirements. The EUA pathway authorized use of the products for the duration of the emergency with further regulatory clearance and FDA approval required upon the conclusion of the emergency. The EUA process was applicable to many product types in the fight against COVID-19, not just for ventilators [9]. Relative to mechanical ventilators, the FDA provided explicit guidance for engineering design in the Emergency Use Ventilator document [10], as detailed in Table 1. With this guidance and the awareness that the typical supply chains might be pushed beyond capacity for ventilator-specific parts, we made design choices to facilitate the build of a functional, rapidly deployable, cost effective mechanical ventilator. Arguably, the single most important decision was to power the ventilator with gases available in hospitals and use time-cycled flow interruption for rate and tidal volume. This design decision obviated the need for an internal compressor, pressurizing components, and bellows.

**Table 1:** Emergency use ventilator minimum requirements for treating COVID-19 patients according to (AAMI, 2020).

Emergency Use Authorization Ventilator Recommendations	
EUA Property	Recommendation
FiO_2_	21% (ambient) to 95% of the source oxygen concentration input (*in no more than 10% steps*)
PEEP	5 to 20 cmH_2_O (*in no more than 5cmH*_*2*_*O steps*)
I:E ratio	1:2 (*preferable adjustable from 1*:*1 to 1*:*3*)
Mandatory Ventilation Mode	Respiratory rate from 10 to 30 inflations/min (*in step of no more than 2 inflations/min*)
Tidal Volume	350 to 450 mL +/- 10% (*in no more than steps of 50mL*, *preferably a lower range of 250mL and an upper range of 600mL or 80mL*)
Inspiratory Pressure Limit	15 to 40 cmH_2_O (*in steps of no more than 5 cmH*_*2*_*O*)
Alarm Conditions	• Gas or electricity supply failure
	• Ventilator switched off while in mandatory mode
	• Inspiratory airway pressure exceeded.
	• Inspiratory and PEEP pressure not achieved
	• Tidal volume not achieved or exceeded
Visual Indicators (Current setting and delivered state)	• Inspiratory pressure
	• Tidal volume
	• Breath rate
	• PEEP
	• FiO_2_
	• Ventilation mode

The FDA offered guidance on the creation of 2 distinct ventilator categories. Specifically, the EUA offered guidance on *Emergency Resuscitators*, devices that provide positive pressure via mask or nasal interface, and Emergency Ventilators. Our ventilator, termed the O2U ventilator, falls under the *Emergency Ventilator* category which entails the capacity to deliver time-cycled, positive pressure breaths, limited by either pressure or volume [10]. The design and testing process for the O2U ventilator is outlined presently.

The primary gas delivery power source for the O2U ventilator is from the pressurized medical gases which are plumbed into the hospital and subsequently into the ventilators [11]. The goal for this ventilator was a design that would permit rapid manufacture of relatively inexpensive ventilators that could withstand long-term storage yet be rapidly deployed to the patient care arena and still reliably deliver time-cycled, precise gas volumes to patients. A secondary goal was the capacity to support a spontaneously breathing patient via mask, nasal prongs or an endotracheal tube as well as an intubated patient with profound respiratory failure incapable of breathing spontaneously. This strategy allows a single ventilator to move with a patient through all phases of hospitalization which is especially important in the case of a highly contagious, fastidious viral pathogen such as COVID-19 [12]. To accomplish these goals, we placed a priority on simplicity of design by minimizing the number of parts, cost, and using readily available parts. The present design allows for ease of use and sufficient ventilatory flexibility to treat patients with mild, moderate, or severe respiratory disease. The user interface is designed such that a provider familiar with fundamental principles of respiratory physiology and mechanical ventilation would be readily able to manage the device to facilitate rapid deployment and ready adoption even when respiratory therapy, nursing and medical personnel are relatively unfamiliar with the O2U ventilator.

## Materials and methods

### Study design philosophy

This represents the first time that the FDA has implemented an Emergency Use Authorization (EUA) for ventilators. The FDA streamlined the traditional 510(k) pathway for ventilators as there were several tests that would normally take a significant amount of time (and money). These were predominantly biocompatibility tests. Examples of these were:

- Permitting the use of known (approved) materials without requiring biocompatibility testing.
- A reduction of the electrical safety requirements the EUA ventilators need to meet to those which are most important to patient and user safety and functionality.
- Allowing risk analyses and materials analysis to fill the gap left by a lack of testing.

Continued usage of EUA ventilators after the emergency period concludes will entail completion of all 510k-required testing and subsequent clearance from the FDA.

Our design was unique from the outset as it was always intended to exist after the EUA phase had passed.

### O2U ventilator design

Most ventilators offer pressure-controlled or volume-controlled ventilation. This requires the use of a closed feedback loop in the case of pressure-controlled, or at least one flow sensor in the volume-controlled case. The O2U ventilator relied on a known flow rate entering the system and control of the valve timings. The pressure-limited, time-cycled design included continuous monitoring of the pressures to detect any leaks or obstructions that could risk patient or device safety.

**Fig 1:**
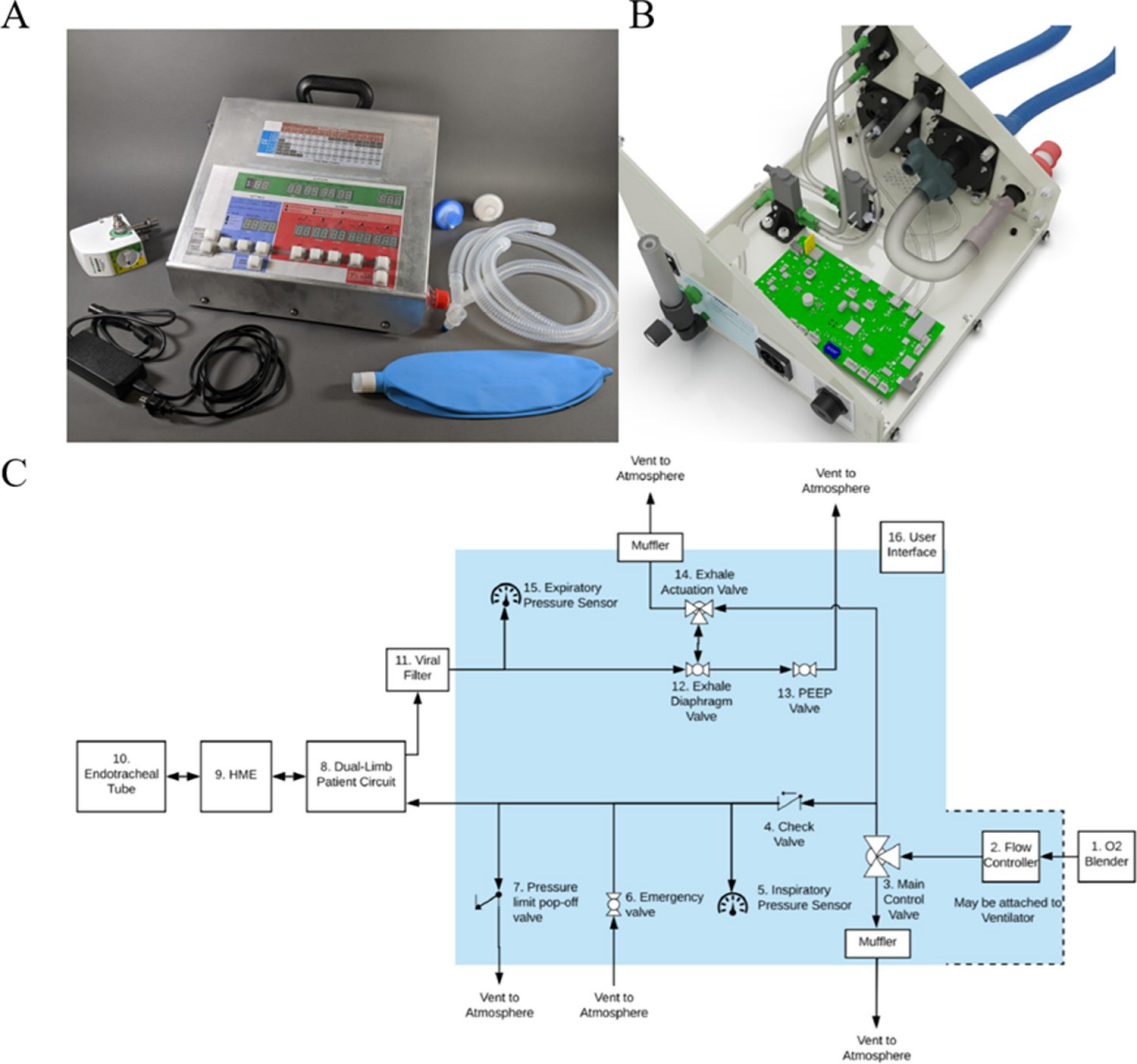
Design of the O2U ventilator. A prototype of the new O2U ventilator, designed and built in the early months of the COVID-19 pandemic shown with accessories (A), the internal construction (B) and the prototype schematic (C) showing the gas pathway internal components (within the shaded region) and the relevant accessories and component required (non-shaded).

The ventilator was connected to pressurized sources of air and oxygen gas. The desired fraction of inspired oxygen (FiO_2_) was mixed with medical-grade air prior to entering the ventilator by means of a gas mixer. During non-invasive operation modes this gas mixture was allowed to pass continuously through the device, entering and exiting the patient breathing circuit, operating in the same manner as other CPAP or High Flow Nasal Cannula systems. Inspiratory and expiratory valves were kept open in this state and the system monitored the pressures in cases of leaks or blockages or any other risks to patient and device safety. For invasive ventilation modes, either assisted or mandatory, the inspiratory and expiratory valves were used to allow or prevent the passage of incoming and outgoing gas, respectively, to enforce the respiration cycle within the patient. To overcome the lack of availability in flow-measuring sensors, the flow rate was set on the ventilator using a manual control valve, also known as a Thorpe Tube, and the inspiratory time was set such that a known volume of gas was delivered during each inspiration phase. The input Thorpe tube regulated the pressure from wall pressures of typically 50 psi down to 1–3 psi, the regular range for patient breathing circuits. A specific tidal volume could then be delivered by knowing the flow rate and adjusting the inspiratory time such that the tidal volume V_T_ could be calculated simply as V_T_ = (Flow rate x Inspiratory time) where the operator could determine, for a user-set flow rate and inspiratory time, the delivered flow volume. Volume calculations are still important for patient care, so in addition to this table for setting the desired delivered volume, a spirometer-based expiratory volume sensor was used on the expiration side of the circuit to measure exhaled volumes. This spirometer-based volume sensor was based on the work of Edmunds et al. [13], whose work was aimed to help measure gas volumes in ventilators for the COVID-19 pandemic. The O2U ventilator prototype is shown in Fig 1.

In comparison to other ventilators currently on the market, the O2U design results in fewer complex components and hence a large reduction in parts cost. The O2U ventilator contains 118 components, whereas the ServoU (Siemens Co.) contains an order of magnitude more component pieces. This component count difference results in an overall cost of build for the O2U ventilator at $1000 USD, compared to the 5,000–40,000 USD cost for conventional ventilators [14]. The main reason for this drastic difference is that the O2U ventilator was designed specifically to meet the FDA’s emergency ventilator guidance and thus provide targeted functionality to treat COVID-19 patients. The user interface for the O2U ventilator includes setting the flow rate control with a Thorpe flow tube to control rate on the exterior of the device. The tidal volume delivered to the patient is the result of the inspiratory time and flow rate. The device includes a table that facilitates determination of the tidal volume. The inspiratory time can be set in 0.2 second increments ranging from 0.x to 1.6 seconds. Respiratory rate is set by the operator and is displayed as breaths per minute. The ventilatory mode is set by the operator with a display indicating mode (CPAP or Intermittent Mandatory Ventilation). The inspiratory to expiratory ratio, peak inspiratory pressure, set tidal volume, and exhaled gas volume are digitally displayed for each breath. PEEP is controlled by a manual value on the side of the ventilator using a diaphragm valve. Alarms, as mandated by the FDA’s emergency guidance documentation, for high pressure and low tidal volume and pressure are included in the device. To deliver this gas mixture to the patient, the O2U ventilator allows for a standard patient breathing circuit to be connected using the standard 22mm interface, along with a standard, external heat-moister exchange device.

### Testing the O2U ventilator

As previously stated, the goal of the O2U ventilator was to simplify the process of respiratory care in the event of a pandemic. This meant a simple to use device was needed, with minimal features, but sufficient fidelity between the input settings and ventilator performance. To demonstrate this, a number of bench-top tests, designed to fulfil the requirements of the EUA, and an animal test were conducted to observe the flexibility of this ventilator in the cases where it was designed to be used. While the EUA did not require any animal testing, this animal test, performed on a 65 kg adult pig, described in Table 2, was used to highlight the performance of the O2U ventilator and any potential risk of damaging normal lungs.

**Table 2:** Ventilator parameters used for the benchtop testing as required for FDA EUA ventilator requirements as well as the animal ventilation test showing the phases of simulated ventilation treatment.

Parameters for Benchtop and Animal Testing				
Test/Phase	PEEP (cmH_2_O)	Breath Rate (breaths/min)	Tidal Volume (mL)	iTime (s)
**Benchtop Tests**				
PEEP Test	5 | 15	10	500	1
Breath Rate Test	5	10 | 15 | 20	500	1
Tidal Volume Test	5	10	300 | 350 | 400 | 450 | 500	1
iTime Test	5	10	500	1.0 | 1.6
**Animal Protocol Phases**				
Baseline	5	10	650	1.2
Hyperventilation	5	10	850	1.6
Hypoventilation	5	10	440	0.8
Baseline/Recovery	5	10	650	1.2

The focus of the EUA requirements was to ensure ventilators would be effective in the treatment of COVID-19 patients. As such, the following variables, relevant to COVID-19 treatment, were tested:

- *Positive End-Expiratory Pressure (PEEP)*: to ensure that the lung always has some positive pressure inside so that the gas exchange units of the lung do not collapse during exhalation. A small amount of PEEP is often used to protect the lungs.
- *Breath Rate (breaths/min delivered)*: contingent on patient condition, age, lung disease, and overall acid-base status, the number of breaths per minute will vary.
- *Tidal Volume*: Lung volumes typically relate to overall body size and hence the tidal volume (the total volume that the ventilator sends during a time-limited inspiration) needs to be variable to treat patients of different size and type of lung pathology.
- *Inspiratory Time (similar to I*:*E ratio)*: based on the breath rate, a fixed amount of time is placed between breaths, this can be split into inspiration and exhalation phases at a ratio, normally more time is given for exhalation as the lungs contract slowly, resulting in a low expiratory flow rate compared to the ventilator driven inspiratory flow rate.

Bench top tests were conducted using a Michigan Instruments test lung. The protocols for these parameter sweeps are outlined in Table 2. The animal test was conducted with an adult pig, the protocol of which is outlined in Table 2. The protocol was approved by the Administrative Panel on Laboratory Animal Care at Stanford University (Protocol Number: 33793). All surgery was performed under anesthesia, and all efforts were made to minimize suffering.

Data for both the test lung testing and animal testing was gathered with two TSI 5320–2 Advanced Gas Mass Flowmeters, placed at the ends of the inspiratory and expiratory lungs.

### Data and statistical analysis

Pressure and flow data from the TSI 5320–2 Advanced Gas Mass Flowmeters were analyzed using Python 3.7 and Jupyter notebooks. Volume was calculated by integrating the flow values to derive minute ventilation and all other volume values. Data was obtained from both the inspiratory and expiratory limbs of the circuit to measure the delivered and exhaled volumes, PIP and PEEP, respectively.

## Results

### EUA-guided bench top testing for ventilator specifications

**Fig 2:**
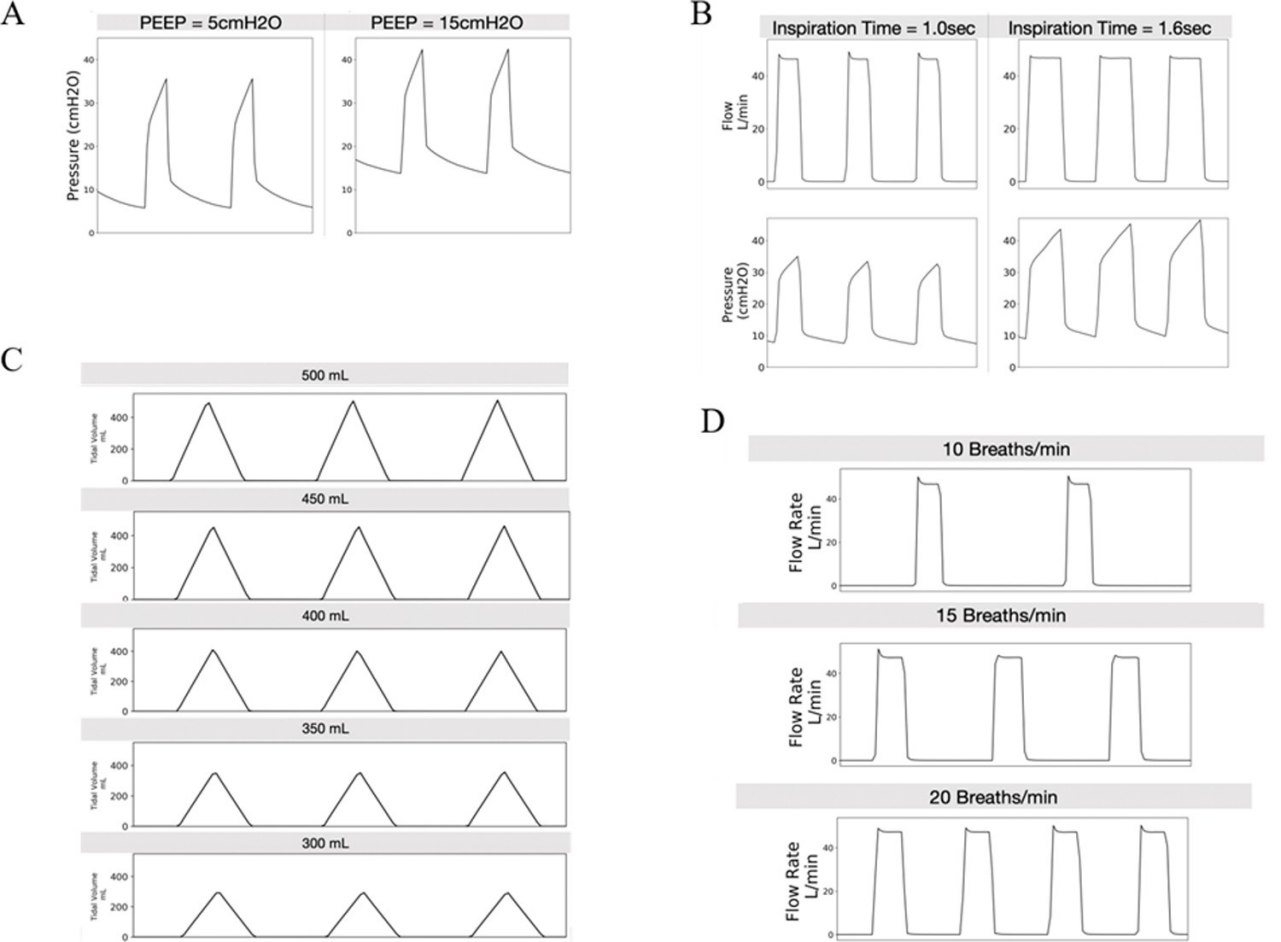
Bench test results of the O2U ventilator. (A) Positive end-expiratory pressure (PEEP) as a function of time as measured on the benchtop test setup for the O2U ventilator (left) PEEP at 5 cmH_2_O and (right) 15 cmH2O set via an external diaphragm valve on the O2U ventilator, x-axis is time. (B) Inspiration time is the duration of time that the gas is delivered to the patient. This is important to be able to vary to manage different lung care strategies and ventilation parameters. Shown here is the difference between 1.0 s and 1.6 s on the dynamic effects on the flow and PEEP pressure signals, x-axis is time. (C) Tidal volume, or the volume delivered to the patient is a critical variable in patient care to ensure adequate oxygenation while not over pressurizing the lungs. The O2U ventilator is capable of providing greater than the FDA-required range to manage the majority of COVID-19 patients. This range is shown in 50 mL increments from 300 mL to 500 mL, x-axis is time. (D) Breath rate (and inspiratory time) controls the minute volume of gas (L/min) supplied to the patient. Control of the breath rate is essential for patient care as this varies based on patient age and underlying conditions and can often change during ventilation. These graphs show 10 (top), 15 (mid), and 20 (bottom) breaths per minute as set by the UI of the O2U ventilator for a flow rate of 50 L/min, x-axis is time.

Data was recorded on the TSI flow meters and processed as comma separated value text files. These files were then post-processed using custom Python scripts to analyze the flow data. Flow of gasses were recorded, along with the pressure. These pressures were measured as absolute values and atmospheric pressure was measured during testing to calculate the gauge pressure, measured in cmH_2_O. All of the tests are listed in Table 2. The analyzed data is shown in Fig 2 for positive end-expiratory pressure (PEEP), breathing rate, tidal volume, and inspiratory time. Data was recorded over several breaths for each test, representative plots are shown here for clarity.

### Porcine ventilation experiment for ventilator treatment testing

During mechanical ventilation (MV), lung injury can occur, especially in the context of respiratory failure. High tidal volumes, high driving pressure, and atelectasis are injurious to the lung and can even lead to damage in end-organs such as the kidney or intestine [15]. In the context of acute injury, ventilatory strategies designed to protect the lung include limiting tidal volumes to 3–6 cc/kg, minimizing the driving pressure, and delivering positive end expiratory pressure to prevent lung collapse [16]. The O2U ventilator was rigorously tested to ensure that MV would occur reliably, effectively, and safely.

**Fig 3:**
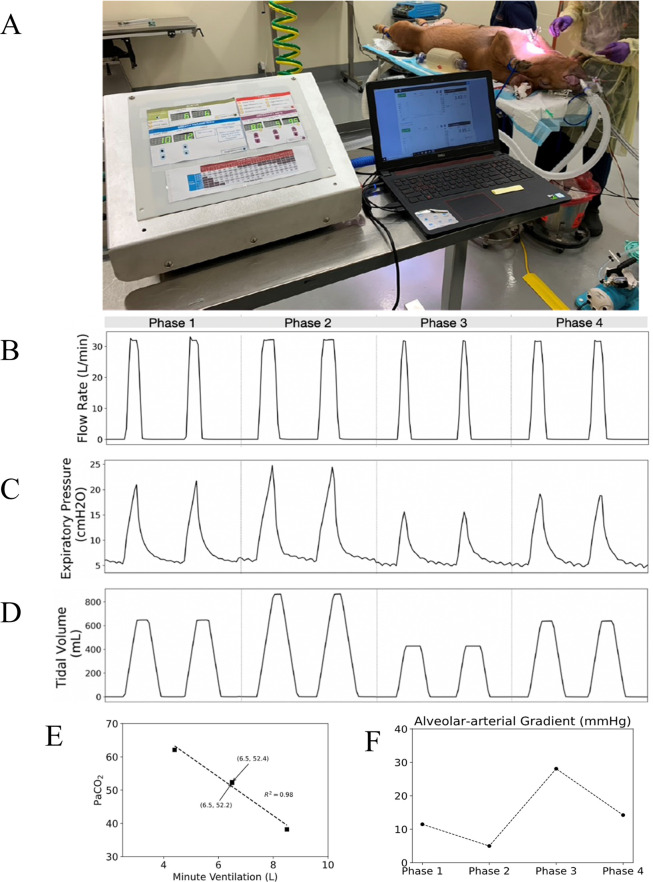
Ventilator and physiology results from the animal test. (A) The O2U ventilator performing mandatory ventilation on a sedated and intubated porcine subject. (B) Flow rate recordings during the different phases of the animal test, while the incoming flow rate, set on the flowmeter, was maintained at 33 L/min with each phase the inspiration time was changed to change the tidal volume, changing the width of the flow peaks. (C) The PEEP was maintained constant at 5 cmH_2_O during the test though the peak expiratory pressure measurements changed according to the current simulated ventilation treatment phase. (D) The tidal volume was the key controlled variable in the animal test with each phase targeting values to correspond to different desired physiological responses, these responses were measured in Table 3. (E) The relationship between the Minute Ventilation and the PaCO_2_ showing the linear inverse relationship. (F) The A-a gradient during the different phases of the experiment.

A porcine study was conducted with the O2U ventilator to ensure fidelity between the ventilation parameters set on the O2U ventilator and the actual ventilation delivered to the animal. Historically, pigs have been employed as an effective large animal model for ventilation [17, 18], owing to similarities in lung architecture, tidal volumes, and respiratory rate. The protocol for the animal test (Table 2) was designed to test the dynamic range of the O2U ventilator. The animal was a 65 kg female pig with normal lungs. Arterial and venous blood samples were drawn from the animal at 15-minute intervals to measure ventilation, oxygenation, and acid-base status. Initially O2U ventilator settings included a tidal volume of 10 cc/kg or 650 cc, a rate of 10 breaths/minute, and an inspiratory time of 1.2 seconds. To prevent atelectasis, PEEP was set at 5 cmH_2_O. PEEP was controlled by a separate control on the expiratory limb of the device (Fig 1). After establishing a stable baseline for 15 minutes of ventilation, minute ventilation was decreased by lowering the tidal volume to 6.46cc/kg or 420 cc for 15 minutes. Given that minute ventilation decreased by 30.7%, we anticipated a proportionate increase in PaCO_2_. After 15 minutes of relative hypoventilation, PaCO_2_ increased to 62.1 mmHg, or by 18.9%. Next, the minute ventilation was increased to 850 cc, an increase of 30.7% from the baseline. After 15 minutes of hyperventilation, PaCO_2_ decreased to 38.2 mmHg, or by 36.6%. Given that PaCO_2_ is inversely and linearly proportional to minute ventilation the observed increase and decreases are consistent with fidelity between the set and delivered minute ventilation [19]. In each experimental condition, PaCO_2_ and minute ventilation were well correlated (Fig 3 and Table 3). Data on the flow and pressure on the inspiratory side was measured using the same TSI flow meter used for the bench-top testing. Flow, pressure, and delivered volume for each phase listed in Table 2 are also shown in Fig 3.

**Table 3:** Measurement of blood gasses of the animal subject during the phases of the ventilation treatment.

Animal Physiology During Testing					
Animal Protocol Phase	V_T_ (mL)	pCO_2_ (mmHg)	pO_2_ (mmHg)	pH	%O_2_
**Arterial Blood Gasses**					
Baseline	650	52.2	73	7.396	94
Hyperventilation	850	38.2	97	7.547	98
Hypoventilation	440	62.1	44	7.357	76
Baseline/Recovery	650	52.4	70	7.419	94
**Venous Blood Gasses**					
Hyperventilation	850	43.4	48	7.488	86
Hypoventilation	440	66.2	38	7.33	66
Baseline/Recovery	650	56.5	46	7.4	80

### Porcine lung x-rays and histology

**Fig 4:**
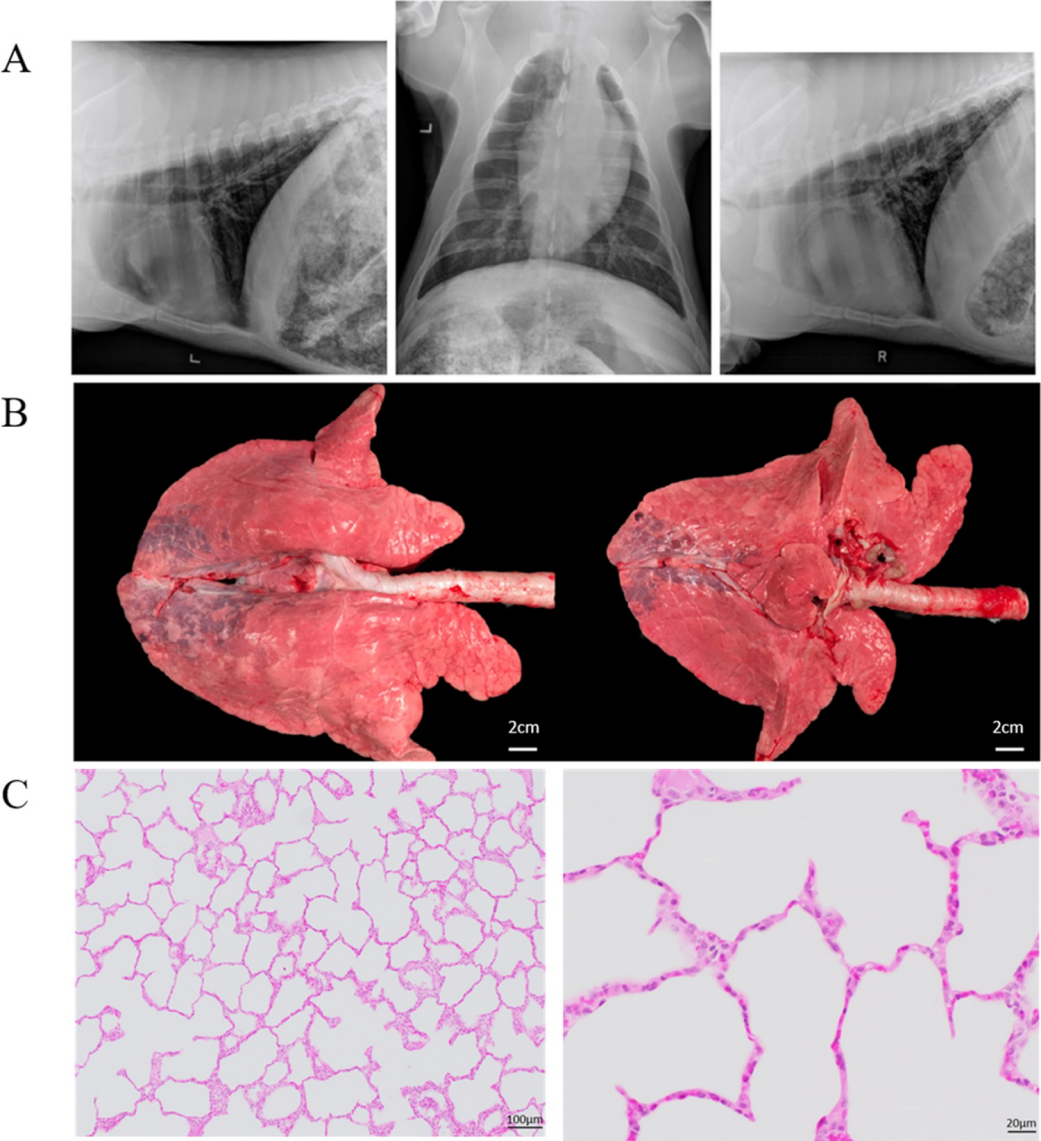
Imaging and histology from the animal experiment. (A) X-Ray images of the animal’s lungs after the ventilator test showing the (left) left lateral thorax, (middle) VD thorax, and (right) right lateral thorax. (B) Images of the animal’s lungs after the experiment. Note the areas of injured lung in the dependent regions of the lung (C) Histology of the lungs at (left) 10x and (right) 40x magnification. Low-magnification (10x) photomicrograph showing the alveolar duct and the associated alveoli. Note the absence of fluid or proteinaceous debris in the alveoli. High-magnification (40x) photomicrograph of lung after mechanical ventilation demonstrating well preserved alveolar structures. The alveolar septa are very thin and consist of flattened alveolar epithelium (pneumocytes) and delicate capillaries.

In addition to the blood gasses measured during the experiment, after ventilation, thoracic radiographs were obtained. Following euthanasia, lungs were inspected, and tissue obtained for microscopic evaluation. Radiographs demonstrated well preserved lung volumes, no hyperinflation but some evidence of increased opacification in the dependent lung regions. Gross inspection was consistent with the radiographs as erythema and venous congestion was apparent in the most caudal and dorsal regions of the lung, which may have been positional as the pig was in dorsal recumbency during the procedure. Histologic evaluation of the lung revealed well preserved alveolar structures, no septal thickening, and no fluid or proteinaceous debris in the airspaces (Fig 4). Microscopic evaluation of the lung tissue did not reveal significant injury. These results provide evidence that the ventilator can deliver sufficient positive pressure ventilation to the lung for a circumscribed time interval (3 hours), without causing injury. Even though lung injury can present after only 20 minutes of ventilation in some murine models [20], the present design does not permit a definitive conclusion surrounding the safety of the present ventilator over an extended time interval. Histologic evaluation of the dependent areas was not performed.

## Discussion

The present work demonstrates the viability and efficacy of a rapidly deployable, low-cost, highly functional ventilator that can be used when demand for ventilators exceeds supply, such as during a pandemic. This design employed a minimal component philosophy, utilizing the pressurized, medical grade, gasses to power gas delivery. The specific tidal volume, rate, and inspiratory time were controlled by valves, with PEEP controlled by a separate control on the expiratory limb of the device (Fig 1). Overall design efficacy and required features were fully compliant with the FDA EUA requirements (see Table 1 for the recommendations and Fig 2 for the measured responses). The FDA recognized the design efficacy and performance of the O2U ventilator and awarded EUA approval in April 2021. This design strategy, especially the notion of powering the ventilator with the pressurized gasses that are available at every accredited hospital in North America, and the overwhelming majority of inpatient health care facilities worldwide, holds the promise of previously unavailable flexibility relative to matching mechanical ventilator supply and demand.

Use of a preclinical porcine model permitted rigorous testing of the device relative to efficacy and safety. The high correlation between minute ventilation input parameters and changes in blood gases in terms of both PaCO_2_ and acid-base status represents strong evidence of fidelity between desired and delivered ventilatory parameters. Moreover, the relatively preserved, albeit imperfectly so, lung volumes demonstrate that the O2U ventilator delivered PEEP throughout the ventilatory cycle and thereby prevented atelectasis in all but the most dependent lung regions. It is likely that a more generous PEEP strategy might have mitigated the dependent atelectasis. The well-preserved lung histology suggests that the O2U ventilator performed well relative to both tidal volume delivery and pressure limits. Important limitations on the data from animal testing include the relatively short time interval of the study and the absence of a lung injury model. Microscopic evaluation of the lung tissue did not reveal significant injury. These results provide evidence that the ventilator can deliver sufficient positive pressure ventilation to the lung for a circumscribed time interval (3 hours), without causing injury. Even though lung injury can present after only 20 minutes of ventilation in some murine models, the present design does not permit a definitive conclusion surrounding the safety of the present ventilator over an extended time interval. Testing the O2U ventilator in a preclinical lung injury model will provide still more information regarding the capacity to facilitate oxygenation when the Alveolar-arteriolar gradient (A-a O_2_) gradient is compromised.

While the results of the testing and the animal subject were very promising, and sufficient to qualify for FDA Emergency Use authorization, there are still some limitations of the proposed design and the presented study. Ideally, for a more thorough and lengthier study of the use of the design in large animals, additional experiments would be conducted. While not required for the FDA, or present in the works of other competing designs, additional experiments would allow for a better understanding of the O2U ventilator function under different physiological conditions. Testing on both healthy and unhealthy subjects would be of value to better assess ventilator function in the context of lung injury, the condition wherein the O2U ventilator would likely be used. As the device has now received FDA authorization, human data will be extremely valuable to ensure efficacy of the O2U ventilator in the clinical arena. The O2U ventilator design, while intended to provide a straightforward interface, possesses limited flexibility relative to conventional ventilators. The reduction in component count for the O2U ventilator is motivated, to a significant degree, by use of an external source of pressurized, premixed, medical grade gas. While this is something readily available in hospitals and other health care facilities, this precludes use of the O2U ventilator in the field. The respiratory cycle of the ventilator includes, at the outset, closure of the inspiratory valve. With the onset of inspiration, the valve is opened and pressurized gas flow unimpeded to the patient until the inspiration time is complete. At the end of the inspiratory time, the inhalation valve is closed and the expiratory value is opened and passive recoil of the lung and patient chest wall permits the patient to exhale. The respiratory cycle of the ventilator includes, at the outset, closure of the inspiratory valve. With the onset of inspiration, the valve is opened and pressurized gas flow unimpeded to the patient until the inspiration time is complete. At the end of the inspiratory time, the inhalation valve is closed, and the expiratory value is opened and passive recoil of the lung and patient chest wall permits the patient to exhale. Effective ventilation requires, at a minimum that the pressure of the gas source be greater than atmospheric pressure. This method of utilizing the pre-pressurized gas is different to the traditional approach whereby a gas mixture is provided to a chamber in the ventilator and compressor within the ventilator’s design delivers the gas to the patient breathing circuit to a predefined pressure. Consequently, this device can be used as a transport ventilator in the presence of a portable supply of pressurized air and oxygen with sufficient volume to provide a continuous supply of medical-grade gasses. Further, given the focus on simplicity of design, a back-up power supply is not included in the present design.

Finally, the current firmware of the O2U ventilator supports mandatory, CPAP, and basic SIMV modes of ventilation. While these modes are extremely useful to support most patients with lung disease, the ability to ventilate using more complex, automated ventilation modes, if enabled, would further improve the efficacy and versatility of the design, but also increase complexity and expense.

Among the limitations of the experiment performed was the absence of humidification. The O2U ventilator, like most ventilators, will rely on external devices. Though ventilation for short periods of time without humidification can be effective, for extended ventilation with the O2U ventilator, or any other mechanical ventilator, use of humidification, to prevent drying of secretions, retention of secretions, blockage of the endotracheal tube, and epithelial injury [21] is necessary. The ventilator design is further limited by the inability to either directly measure or infer the plateau pressure. However, plateau pressure will be less than peak pressure, so providers can modify rate and tidal volume to limit peak pressure and thereby mitigate potential lung injury. While the O2U is designed to function and deliver the ordered tidal volume for every breath, provided that sufficient pressure above atmospheric pressure, the present experimental series did not specifically address potential variability in tidal volume when the driving pressure approaches a lower functional limit.

With the pandemic continuing, hospitals and medical professionals now have a far greater understanding of how to manage those infected. Early guidance at the outset of the COVID-19 crisis suggested that early initiation of mechanical ventilation would improve outcomes and mitigate the risk of dissemination of the virus [8]. With increasing insight into the natural history and pathophysiology of COVID-19 related respiratory disease, guidance has changed. Use of non-invasive strategies for oxygen delivery, including high flow humidified oxygen, and positive pressure ventilation delivered via face mask as continuous positive airway pressure (CPAP) or dual level ventilation with both PEEP and delivery of a tidal breath until a peak inspiratory pressure (PIP) is reached, are employed until and unless respiratory status mandates intubation and mechanical ventilation [22]. A benefit of the O2U ventilator design is the capacity to deliver both non-invasive and invasive respiratory support in modes that include CPAP, continuous delivery of oxygen via nasal cannula, bi-level ventilation, and mechanical ventilation. Thus, the O2U ventilator can remain with an individual patient throughout a course of illness, thereby diminishing the risk viral spread. Given the short timelines in the early stages of the pandemic, the FDA provided critically important requirements for ventilator design. However, now that some time has passed and more thought has been given generally, some more potential risks that were not covered in the initial FDA guidance can and should be considered. An important consideration in the O2U design is the use of pressurized gas to power inspiratory flow. Thus, variation in airflow will have a significant impact on the clinical performance of the O2U ventilator.

Given uneven access to the COVID-19 vaccine, the pandemic continues to cause illness and death in many parts of the world. Hence, ventilators remain in short supply wherever the disease surges. Likely, with the emergence of new pathogens, the episodic increase in demand for ventilators will persist for decades to come. In North America, the pre-pandemic ventilator rate of production was approximately 700 new ventilators per week [22], but swelled to 7000 during the Spring of 2020 as Ford, GM, and EUA-guided ventilators become available to meet the need. Nonetheless, Wells et al. [23] estimated both the number of available invasive ventilators (~70,000) and non-invasive ventilators (~18,000) for ICU patients was dwarfed by the need for invasive and non-invasive ventilators, 110,000 and 95,000, respectively. In anticipation of the increased need, the US Government enacted the Defense Production Act to catalyze the production 200,000 ventilators from nearly a dozen manufactures [24]. Branson et al., concluded that new stockpile ventilators should be designed for much faster end-user education and deployment, much as like the current O2U ventilator. A physiologically based interface simplifies the design to maximize the number of caregivers that can safely and effectively manage these ventilators without time-intensive training. A further strength of the O2U ventilator is that it meets the current FDA standards. Further, testing the O2U ventilator in a preclinical animal model provides a level of confidence that the O2U ventilator will function as designed in a clinical setting. The design focus on fundamental principles of respiratory physiology will allow for physicians, respiratory therapists, and nursing personnel to readily apply the O2U ventilator to patients without device specific training.

## Conclusion

In this study we presented a new, versatile, rapid-response based design ventilator for the current COVID-19 pandemic. Our design has been authorized under the FDA’s Emergency Use requirements. The relatively straightforward, physiologically well-grounded design will permit ready deployment into the clinical arena. The cost and simplicity of the design promises to motivate production at scale. These properties may enable the O2U ventilator to fill a large gap in our current healthcare armamentarium, specifically a scalable, inexpensive, and highly functional mechanical ventilator. The O2U ventilator is especially well positioned to address the marked divergence of supply and demand that has happened, and will continue to occur, in large scale pandemics worldwide.
